# Comparison of the bacterial communities in feces from wild versus housed sables (*Martes zibellina*) by high-throughput sequence analysis of the bacterial 16S rRNA gene

**DOI:** 10.1186/s13568-016-0254-4

**Published:** 2016-10-13

**Authors:** Yu Guan, Honghai Zhang, Xiaodong Gao, Shuai Shang, Xiaoyang Wu, Jun Chen, Wei Zhang, Weihua Zhang, Mingsheng Jiang, Baohong Zhang, Peng Chen

**Affiliations:** 1 College of Life Science, Qufu Normal University, Qufu, Shandong China; 2 College of Wildlife Resource, Northeast Forestry University, Harbin, Heilongjiang China; 3 Administrative Bureau of Khan Ma National Nature Reserve, Inner Mongolia, China; 4 Administrative Bureau of Shuang He National Nature Reserve, Heilongjiang, China

**Keywords:** Sable (*Martes zibellina*), 16S rRNA gene, Fecal microbiota, Bacterial diversity

## Abstract

The composition of mammalian intestinal microflora is related to many environmental and geographical factors, and it plays an important role in many aspects such as growth and development. Sequencing data of the bacterial 16S rRNA gene from sable (*Martes zibellina*) samples using next-generation sequencing technology are limited. In our research, 84,116 reads obtained by high-throughput sequencing were analyzed to characterize and compare the intestinal microflora of wild sables and housed sables. *Firmicutes* (31.1 %), *Bacteroidetes* (26.0 %) and *Proteobacteria* (21.5 %) were the three most abundant phyla present in wild sables, whereas *Firmicutes* (55.6 %), *Proteobacteria* (29.1 %) and *Actinobacteria* (6.0 %) were the three predominant phyla present in housed sables. At the phylum level, wild sables exhibited a significant difference in the relative abundances of *Bacteroidetes* and *Actinobacteria*, whereas housed sables only exhibited significant changes in *TM7* at the phylum level, and *Clostridia*, at the class level. The predominance of *Bacteroidetes* in wild sables warrants further research. These results indicate that a sudden change in diet may be a key factor that influences fecal bacterial diversity in mammals.

## Introduction

The mammalian gastrointestinal tract contains a complex microbial community that encompasses trillions of bacteria. In some ways, gut microbiota could be regarded as a separate organ that is composed of 1000–1200 cell types that encode 150-fold more genes than are present in the human genome (Rosenberg et al. 2013). Recent studies have shown that the intestinal microbiota plays an important role in modulating the steady-state balance of the intestine and that alterations in this complex microbial community have been associated with the host age, diet, and health (Tilg and Kaser 2011). For mammals, diet is likely a key factor that influences the bacterial diversity observed between carnivores, omnivores and herbivores (Ley et al. 2008).

The sable *Martes zibellina* (Linnaeus, 1758) is a mustelid species of great interests due to its valuable fur (Numerov 1963). Sables inhabit the region that extends southward to 55°N–60°N latitude in western Siberia and to 42°N in the mountains of eastern Asia (Monakhov 2011). Unfortunately, the rampant international underground trade of sable pelts and the reduction of their habitats have caused this valuable species to be written in the IUCN Red List of Threatened Species in 2008. Facing these worrisome states, the preservation of sables and their habitats becomes extremely grim and urgent. However, much of the previous researches about sable are almost centering on the macro scale ecosystem for its protection. For example, studies published by Zhang and Ma (1999) regarding sable habitat preferences in the winter provided a great deal of information, as well as suggestions, for habitat preservation during tree selection cutting. Bao et al. (2003) and Brzezinski (1994) analyzed changes in sable diets over the course of different seasons and across various districts. These findings informed alterations in dietary and reproductive conditions at large city zoos and fur farms.

Although these results are encouraging, further improvements for sable conservation are necessary. Fortunately, the development of next-generation sequencing facilitates the characterization of complex microbial communities more accurately and rapidly. Therefore, the objectives of our study were to characterize and compare the fecal microbiota of sables between wild and housed sables.

## Materials and methods

### Fecal sample collection

Fecal samples from wild sables (Wild sable 1–3) were collected during December 2014 and from different regions in the Khan Ma National Nature Reserve of Inner Mongolia, China. Heavy snow coverage and low temperature (−30 to −40 °C) kept the feces fresh and clean as much as possible. To prevent the other contaminations that could pollute feces, the wild samples were then preserved in ethyl alcohol in time before they were frozen in refrigerator.

Fecal samples from housed sables (Housed sable 1–14) were collected within a half hour after defecation from Dalian Mingwei Marten Industry Co., Ltd during May 2014. These housed sables were caught from Mo He, Daxinganling Mountains and fed in Dalian for 3 months temporarily. They were fed with a diet that contains fresh fish, eggs and a small percentage of wheat bran, which was similar to the diet of housed minks kept at the same location. Throughout this process, we monitored the health of housed sables and ensured that none of them received antibiotic or probiotic therapy for the past 3 months.

Housed sables in our experiment were caught from Daxinganling Mountains and raised at Dalian Mingwei Marten Industry Co., Ltd. We captured wild sables with the traditional Chinese traps and there were no any harms for sables during the process. All of the methods for catching and feeding this endangered animal were estimated and permitted by The Wild Protection and Nature Reserve Management of the State Forestry Administration of the People’s Republic of China.

All fecal samples were immediately frozen and stored at −80 °C until they were processed.

### DNA extraction

DNA was extracted using the QIAamp® DNA Stool Mini Kit (Qiagen, Hilden, Germany) according to the provided QIAamp® DNA Stool protocol.

### PCR amplification, purification and sequencing

A 16S universal amplicon PCR forward primer (5′-CCTACGGGNGGCWGCAG-3′) and reverse primer (5′-GACTACHVGGGTATCTAATCC-3′) were used to amplify the V3 and V4 regions of the 16S rRNA gene. Polymerase chain reaction was carried out using the following mixture in a final volume of 50 μL: 6 μL of DNA for template, 2 μL of each primer (10 μM), 5 μL of 10× Ex PCR buffer, 4 μL of dNTP (10 mM each), 0.5 μL of BSA, 0.5 μL of Ex Taq DNA polymerase (5 U/μL) and 30 μL of ddH_2_O. Next, DNA was amplified using the following conditions: 3 min at 95 °C for denaturation, followed by 25 cycles of 30 s at 95 °C for denaturation, 30 s at 55 °C for annealing and 30 s at 72 °C for extension, as well as a final extension step at 72 °C for 5 min.

The yield of PCR products was estimated using agarose (2 %) gel electrophoresis, and then the PCR products were purified using the QIAquick® PCR Purification Kit (QIAGEN, Hilden, Germany). After this PCR clean-up step, we followed the Illumina MiSeq protocol to perform the Index PCR and PCR clean-up 2 steps. Next, the products were processed according to the manufacturer’s instructions for the Qubit® dsDNA HS Assay Kit (Invitrogen, Carlsbad CA) and quantified using the Qubit® 2.0 Fluorometer (Invitrogen). Finally, the products were sequenced with an Illumina MiSeq (illumina, USA) according to the manufacturer’s instructions.

### Sequence processing and statistical analysis

After the libraries were filtered to remove 5′ and 3′ overhangs, original sequences were analyzed using MOTHUR (Kozich et al. 2013) to eliminate noise (Quince et al. 2011) and check for chimeras (Edgar et al. 2011) by commands in operation manual. Next, the relative abundances of bacteria were calculated. The 100 % stacked column charts was also generated, which represent the bacteria in the two groups and intra-group at the phylum level. By using the SILVA 16S rRNA reference database, the sequences were assigned into OTUs (operational taxonomic unit) at a 0.03 cutoff level.

The coverage, the inverse Simpson index and the rarefaction curves were also generated using MOTHUR. The rarefaction curves were then analyzed with Microsoft Excel, and the percentage of each phylum, class, order, family and genus between the two groups were compared by *T* test, and 95 % confidence intervals were calculated. To assess the dissimilarity between wild and housed groups, we used the Jclass (Jaccard) index and YC (Yue and Clayton) method at both the phylum and genus level. We also used a phylotype-based approach at the genus level and the same methods to compare the dissimilarity between wild and housed groups as OTUs-based approach.

To assess similarities in bacterial population among all individuals, phylogenetic trees were generated with MOTHUR using both methods mentioned above. TreeView 1.6.6 was used to depict the dendrograms. We also used Mothur to determine if there was a significant difference in the clustering among samples by using the Parsimony test, the weighted UniFrac method, and the unweighted UniFrac method. Finally, to determine whether there was a difference using Mothur’s OTU-based analysis and Phylotype-based analysis, we performed principal coordinate analysis (PCoA) and non-metric multidimensional scaling (NMDS) analysis according to the commands from Mothur.

The original sequence data have been submitted to the NCBI Sequence Read Archive with the following accession number: SRA280882.

## Results

### Relative abundance

**Fig. 1:**
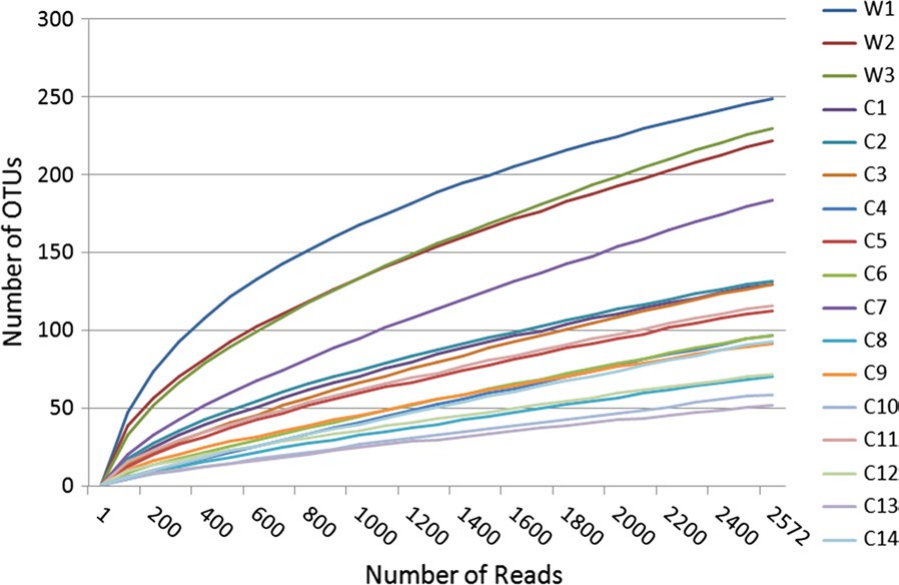
Rarefaction curves. Rarefaction curves, calculated at 3 % dissimilarity, compare the number of reads with the number of operational taxonomic units (OTUs) found in the DNA from the fecal matter of wild sables (W 1–3) and housed sables in fur farms (C 1–14)

A total of 84,116 reads were classified into different OTUs and used to analyze relative abundance and bacterial community diversity of sables. The rarefaction curves were calculated using MOTHUR and plotted in Fig. 1. Due to the rarefaction curves appeared no much fluctuation or growth along with the increasing of the size of our data. Then we compared the curves with the counterpart of other researches and confirmed that the curves had reached the level. So 2572 reads for per sample are sufficient for the following diversity analysis (Table 1).

**Table 1:** Classification of fecal bacteria in wild sables and housed sables

Sable	*Fir*	*Bac*	*Pro*	*Act*	*unc*	*Fus*	*TM7*	*Ten*	*Ver*	*Pla*	*Gem*	Total
W1	21.4	33.9	20.4	18.6	3.2	1.1	0.7	–	–	–	–	3490
W2	47.0	22.4	11.6	10.8	4.9	1.9	0.4	0.7	–	–	–	3623
W3	25.0	21.6	32.6	12.9	1.9	3.0	0.4	0.8	0.8	0.4	–	3252
Mean	31.1	26.0	21.5	14.1	3.3	2.0	0.5	0.5	0.3	0.1	–	10,365
C1	20.1	14.3	59.7	3.2	–	2.6	–	–	–	–	–	3360
C2	28.2	20.9	42.3	3.1	1.8	1.8	–	1.2	–	–	–	3662
C3	26.8	9.4	44.3	12.1	2.7	–	1.3	–	0.7	1.3	1.3	3136
C4	81.7	0.9	8.3	1.8	0.9	2.8	3.7	–	–	–	–	2937
C5	40.0	4.6	30.8	17.7	1.5	3.1	1.5	–	–	–	–	3157
C6	85.0	2.7	4.4	5.3	–	1.8	0.9	–	–	–	–	3102
C7	19.4	3.1	56.5	14.7	1.6	–	3.1	–	0.5	0.5	–	2712
C8	78.4	1.1	13.6	6.8	–	–	–	–	–	–	–	3331
C9	58.3	2.1	35.4	1.0	–	1.0	2.1	–	–	–	–	2719
C10	39.0	–	57.6	–	1.7	1.7	–	–	–	–	–	2572
C11	47.6	3.4	29.0	11.0	2.1	2.1	2.1	–	2.8	–	–	3582
C12	77.0	–	18.4	1.1	1.1	–	2.3	–	–	–	–	3266
C13	93.2	1.7	1.7	1.7	–	–	1.7	–	–	–	–	2983
C14	83.5	3.5	5.2	4.3	0.9	1.7	–	–	–	0.9	–	3240
Mean	55.6	4.8	29.1	6.0	1.0	1.3	1.3	0.1	0.3	0.2	0.1	43,759
Total												54,124

The names of the bacterial phyla are *Firmicutes*, *Bacteroidetes*, *Proteobacteria*, *Actinobacteria*, *unclassified*, candidate group *TM7*, *Tenericutes*, *Verrucomicrobia*, *Planctomycetes*, and *Gemmatimonadetes*

In the fecal samples from wild sables, the most prevalent phylum was *Firmicutes* (31.1 %), followed by *Bacteroidetes* (26.0 %) and *Proteobacteria* (21.5 %). However, it is worth noting that the relatively high abundance of *Firmicutes* may be skewed by wild sable 2. *Bacteroides* was the predominant genus in wild sable 2, followed by *Parabacteroides* and *Blautia*. In contrast, *Barnesiella* and *Bacteroides* were the most common genera in both wild sable 1 and wild sable 3.

*Firmicutes* (55.6 %) were also the most common phylum in fecal matter from housed sables, followed by *Proteobacteria* (29.1 %), *Actinobacteria* (6.0 %) and *Bacteroidetes* (4.8 %). The most common genera included *Clostridium* (4 sables), *Bacteroides* (2 sables), *Psychrobacter* (2 sables), *Pseudomonas*, *SphingomonasTM7*, *Streptococcus*, *Escherichia* and *Lactobacillus*.

Additionally, the relative abundances of *Firmicutes*, *Proteobacteria*, *Fusobacteria* were not significantly different among sables living in disparate environments (P = 0.064, P = 0.393 and P = 0.375, respectively). The relative abundances of *Bacteroidetes* and *Actinobacteria* were significantly higher among wild sables (P = 0.021 and P = 0.044), and the relative abundance of TM7 (P = 0.029) was higher among housed sables. Apart from *Actinobacteria*, *Bacteroidia* and *TM7*, *Alphaproteobacteria* (P = 0.020) and *Clostridia* (P = 0.002) were the Classes that were significantly different between the groups. Similarly, the abundances of order *Clostridiales* (P = 0.02), family *Clostridiaceae* (P = 0.0002) and genus *Clostridium* (P = 0.001) were significantly higher in housed sables.

**Fig. 2:**
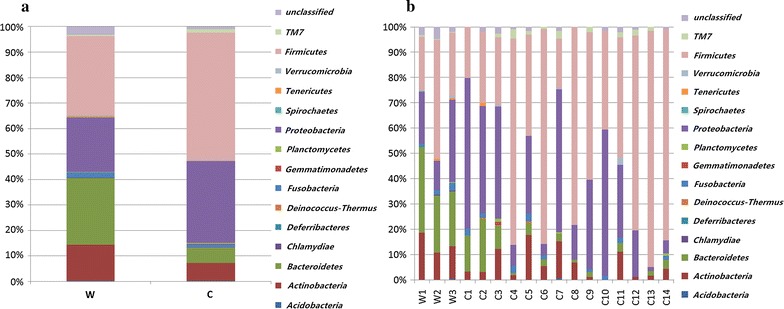
Fecal bacterial population. Overall percentages of bacterial population between two sable groups (**a**) and within the same group (**b**) at the phylum level

The relative abundances of bacterial populations at the phylum level between wild versus housed sables and within the same group are presented in Fig. 2a, b.

### OTU-based analysis

The number of reads, number of OTUs, the inverse Simpson index, coverage and confidence intervals for OTUs are presented in Table 2.

**Table 2:** Total number of sequences, coverage, number of OTUs and inverted Simpson with lower and upper confidence interval limits

Sable	*Fir*	*Bac*	*Pro*	*Act*	*unc*	*Fus*	*TM7*	*Ten*	*Ver*	*Pla*	*Gem*	Total
W1	21.4	33.9	20.4	18.6	3.2	1.1	0.7	–	–	–	–	3490
W2	47.0	22.4	11.6	10.8	4.9	1.9	0.4	0.7	–	–	–	3623
W3	25.0	21.6	32.6	12.9	1.9	3.0	0.4	0.8	0.8	0.4	–	3252
Mean	31.1	26.0	21.5	14.1	3.3	2.0	0.5	0.5	0.3	0.1	–	10,365
C1	20.1	14.3	59.7	3.2	–	2.6	–	–	–	–	–	3360
C2	28.2	20.9	42.3	3.1	1.8	1.8	–	1.2	–	–	–	3662
C3	26.8	9.4	44.3	12.1	2.7	–	1.3	–	0.7	1.3	1.3	3136
C4	81.7	0.9	8.3	1.8	0.9	2.8	3.7	–	–	–	–	2937
C5	40.0	4.6	30.8	17.7	1.5	3.1	1.5	–	–	–	–	3157
C6	85.0	2.7	4.4	5.3	–	1.8	0.9	–	–	–	–	3102
C7	19.4	3.1	56.5	14.7	1.6	–	3.1	–	0.5	0.5	–	2712
C8	78.4	1.1	13.6	6.8	–	–	–	–	–	–	–	3331
C9	58.3	2.1	35.4	1.0	–	1.0	2.1	–	–	–	–	2719
C10	39.0	–	57.6	–	1.7	1.7	–	–	–	–	–	2572
C11	47.6	3.4	29.0	11.0	2.1	2.1	2.1	–	2.8	–	–	3582
C12	77.0	–	18.4	1.1	1.1	–	2.3	–	–	–	–	3266
C13	93.2	1.7	1.7	1.7	–	–	1.7	–	–	–	–	2983
C14	83.5	3.5	5.2	4.3	0.9	1.7	–	–	–	0.9	–	3240
Mean	55.6	4.8	29.1	6.0	1.0	1.3	1.3	0.1	0.3	0.2	0.1	43,759
Total												54,124

**Fig. 3:**
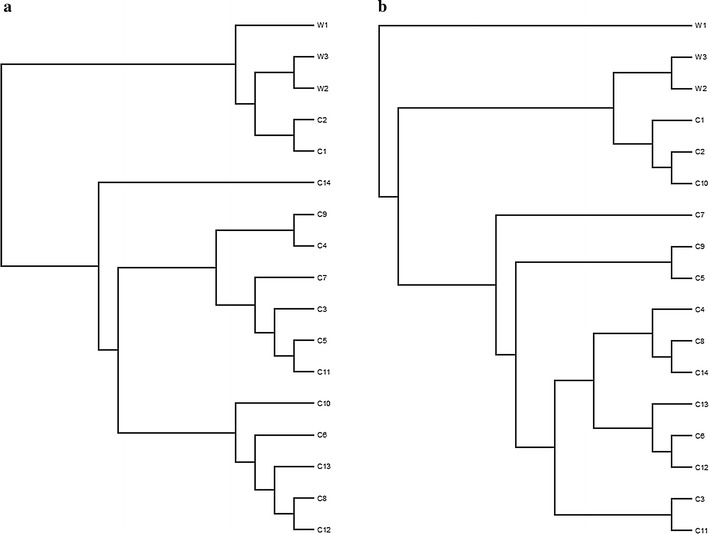
Phylogenetic trees—OTUs approach. Phylogenetic trees comparing the OTUs in the fecal samples of wild sables (w 1–3) and housed sables (C 1–14). The results were calculated by the Jclass index (**a**) and YC measure (**b**)

In accordance with the Miseq MOTHUR protocols, the Jclass and YC calculators were used to generate the phylogenetic trees to visualize the similarity of the OTUs found in fecal samples of wild sables and housed sables. The dendrograms are presented in Fig. 3a, b.

Next, the Parsimony test, weighted UniFrac method and unweighted UniFrac method were used to determine whether the clustering within the tree was statistically significant. However, using both the Jclass (P = 0.126) and the YC (P = 0.128) dissimilarity indices, the results from the Parsimony test indicated that the diversity of bacteria from fecal samples was not significantly different. Because these methods ignore the branch length, we also performed these tests using the weighted UniFrac values and found that the bacterial populations of wild and housed sables were significantly different using both the Jclass (P < 0.001) and YC (P < 0.001) indices. When we used the unweighted UniFrac values for analysis, the Jclass index (P = 0.265) indicated that the two sable groups were not significantly different whereas the YC index (P < 0.001) showed that the two populations were significantly different.

**Fig. 4:**
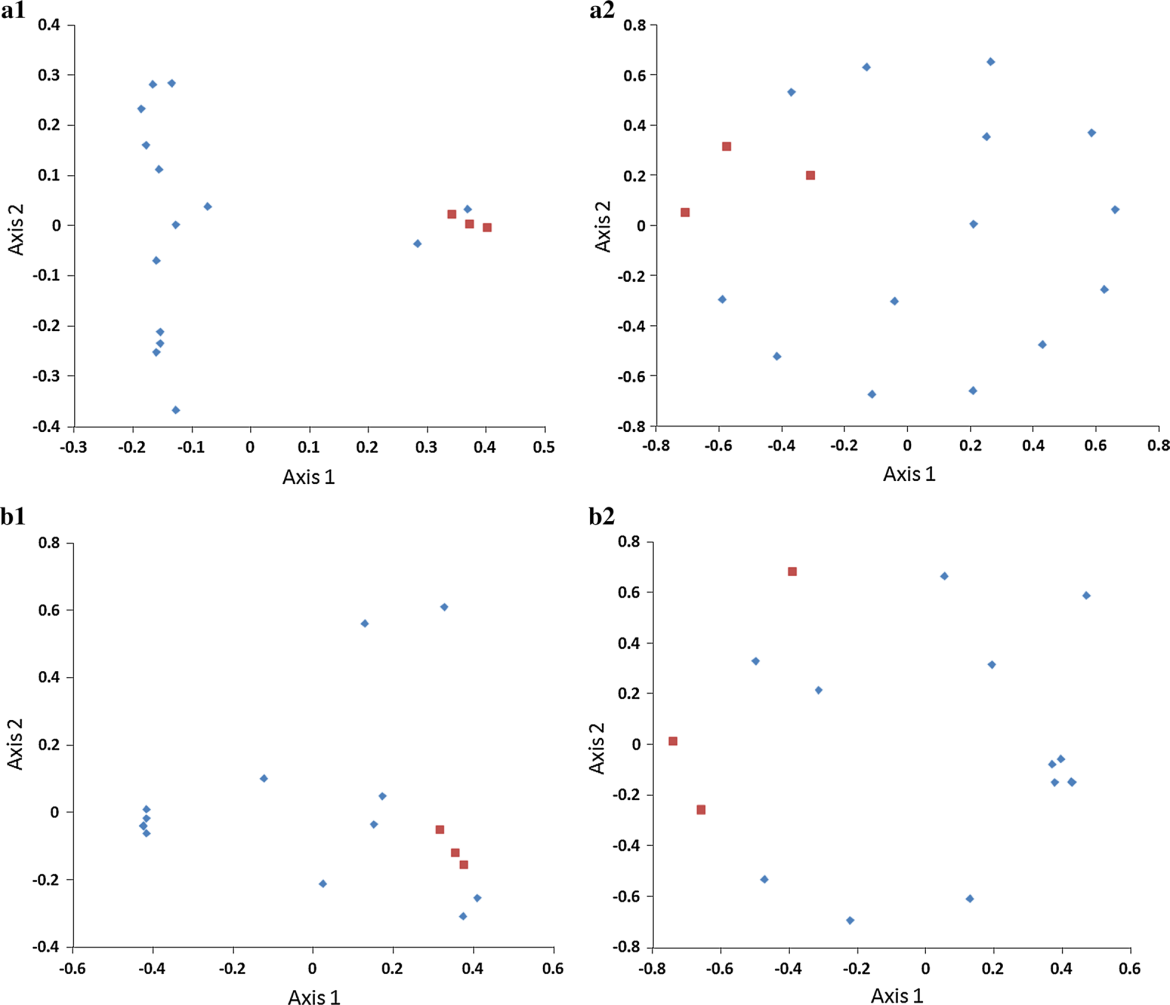
PCoA and NMDS. PCoA (**a1** and **b1**) and NMDS (**a2** and **b2**) correlation analysis of the reads sequenced from the fecal matter of wild sable (*red dots*) and housed sable (*blue dots*). **a1** and **a2** were generated using the Jclass index, **b1** and **b2** were obtained using YC method

Principal coordinate analysis and NMDS analysis with the Jclass index (Fig. 4a1, a2) and the YC values (Fig. 4b1, b2) were conducted with MOTHUR. Using the AMOVA test (P < 0.01), it was clear that the NMDS plots of wild and housed sables were significantly different.

### Phylotype-based analysis

The number of reads, number of OTUs, and the inverse Simpson index, with upper and lower confidence intervals for OTUs are presented in Table 3.

**Table 3:** Total number of sequences, coverage, number of phylotypes and inverted Simpson with lower and upper confidence interval limits

Sable	Total reads	Analyzed reads	Coverage	Phylotypes	Simpson	Lower ci	Upper ci
W1	4919	3490	0.995	53	9.086	8.665	9.549
W2	5050	3623	0.989	90	9.496	8.851	10.244
W3	5818	3252	0.988	98	8.477	7.922	9.116
C1	4720	3360	0.990	66	4.892	4.684	5.119
C2	5018	3662	0.993	58	4.753	4.567	4.955
C3	4803	3136	0.983	78	2.426	2.340	2.519
C4	4606	2937	0.997	21	1.144	1.123	1.168
C5	4795	3157	0.985	72	2.015	1.934	2.103
C6	4879	3102	0.995	24	1.918	1.844	1.998
C7	4669	2712	0.987	78	2.483	2.357	2.624
C8	4844	3331	0.994	22	1.127	1.106	1.148
C9	5289	2719	0.996	31	2.287	2.179	2.386
C10	4588	2572	0.998	12	1.798	1.744	1.855
C11	5077	3582	0.988	64	1.992	1.903	2.090
C12	4900	3266	0.997	20	1.764	1.696	1.837
C13	5172	2983	0.997	12	1.362	1.324	1.403
C14	4969	3240	0.996	21	1.225	1.196	1.255

**Fig. 5:**
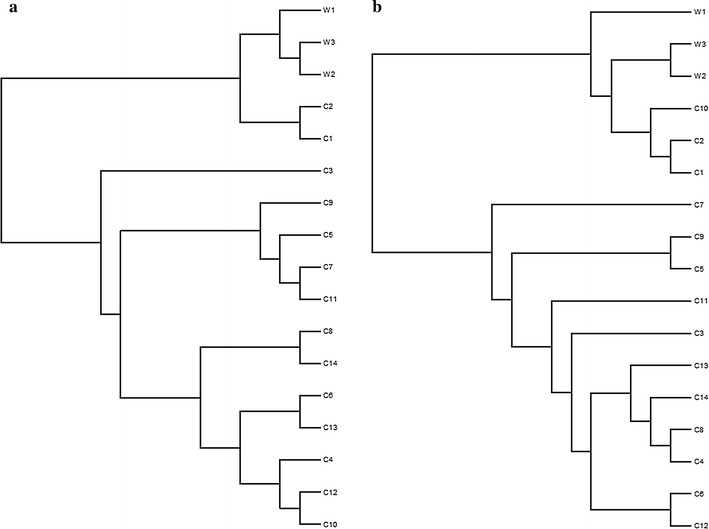
Phylogenetic trees—phylotypes approach. Phylotypic comparison of the fecal samples from wild sables (W 1–3) and housed sables (C 1–14) were depicted using the Jclass (**a**) and the YC (**b**) methods

Mothur was used to generate phylogenetic trees calculated with Jclass and YC indices (Fig. 5a, b). When branch length is ignored, the Parsimony test for both the Jclass (P = 0.002) and YC (P = 0.14) methods indicated that the bacterial populations were not significantly different. However, after taking the branch length into consideration by using the weighted UniFrac, the structure of the communities were significantly different when using both the Jclass (P < 0.001) and YC (P < 0.001) indices. This discrepancy is similar to the results obtained with the weighted UniFrac for the OTU-based approach. When using the unweighted UniFrac, there was no consistent statistical difference between the two groups using the Jclass (P = 0.085) or YC (P = 0.003) indices.

Similar to the OTU-based analysis, AMOVA indicated that the differences observed between wild and housed samples using phylotype-based analysis were statistically significant for both the Jclass (P = 0.004) and YC (P = 0.007) indices.

## Discussion

Sables (*M. zibellina*) are considered to be a flagship species of the Daxinganling Mountains, and China has already included them on its list of protected animals. Due to its enormous economic value and the market demand for its valuable fur, the protection of this unique animal has become an urgent problem that must be addressed. However, there is no any data have been published characterizing or comparing the fecal microbiota of sables by high-throughput sequencing of the bacterial 16S rRNA genes.

This study is an elementary characterization and comparison of bacterial communities in sables that were exposed to different dietary and environmental conditions.

Our results demonstrate that the predominant bacterial phylum in fecal samples from both wild and housed sables was *Firmicutes*, which is consistent with the findings of fecal studies in other mammals such as horses (White et al. 2009) and snow leopards (Zhang et al. 2015). As is typical with omnivorous animals, sables may have limited food options in different environments, particularly during the winter (Xu et al. 1996). The predominance of *Firmicutes* may be connected with feeding habits (Costa et al. 2012) or correlated with a significant change in diet (Middelbos et al. 2010). A dietary preference for berries and nuts during the long and cold winter months may result in a change in the relative abundance of this phylum. The percentage of *Firmicutes* in fecal samples from wild and housed sables was 31.15 % and 55.59 %, respectively. However, the difference between wild and housed sables was not statistically significant (P = 0.064), which may indicate that dietary changes were not the only factor contributing to the obvious alterations in bacterial populations. Indeed, Davenport et al. (2014) reported a seasonal variation in the human gut microbiome, which demonstrates that environmental factors are also an important key to understanding this complex process.

Notably, the structure of bacterial communities present in wild 1 was similar to that in wild 3, and both have relatively high abundances of *Proteobacteria* and *Actinobacteria*. Although Packey and Sartor (2009) and Chang et al. (2008) suggested that *Proteobacteria* are closely correlated with inflammatory bowel disorder (IBD) and *Clostridium difficile* infection, fecal samples found in snow cannot inform us about any illnesses present in the wild sables. Increases in microbial diversity due to a plant-based diet have been linked with *Proteobacteria*, which is consistent with our assumption that dietary changes are responsible for the differences we observed. The intake of saturated fat and animal protein may decrease microbial diversity and cause an increased abundance of *Actinobacteria* (He et al. 2013) which we found to be significantly different between wild and housed sable at the phylum level (P = 0.044). It addition to plants, it is possible that the types of small mammal that were available, such as rats and birds, play a role in the observed difference in bacterial populations. Unfortunately, sables housed at the fur farms were fed a primarily fish-based diet, so there was not enough data for housed sables with a diet rich in small mammals. It would be interesting to compare the bacterial populations of wild and housed sables fed identical diets to assess the effect of different living environments and how they contribute to bacterial diversity in sable fecal samples.

The fecal samples from the three wild sables were collected from nearly identical environments in Daxinganling Mountains because that area is an ideal sable habitat with the sufficient food and space for individual members.

The large differences observed in intestinal bacterial diversity from wild sable 3 were difficult to explain but could be attributed to differences in age (Yatsunenko et al. 2012) or geography (Amanda et al. 2010). Moreover, the difficulty with collecting fecal samples (Ma et al. 1999) and the fact that sables are a protected class in China limited the accuracy of these results. Therefore, the findings in subsequent experiments would be enhanced by data from more wild sable samples. Despite the limited sample size, the data we obtained provided insight about the differences between wild and housed sables and warrant further study.

The relative abundance of *Bacteroidetes* was also significantly higher (P = 0.021) in wild sables (25.97 %) compared with 4.84 % in housed sables. *Bacteroidetes* has been reported to be the most abundant phylum in healthy people (Eckburg et al. 2005), which is consistent with this analysis of wild sables. Turnbaugh et al. (2009a, b) also demonstrated that a decrease in this phylum may be correlated with the obesity. Considering that sables live in complex topography and possess swift responses to danger, it is possible that *Bacteroidetes* play an important role in maintaining a slender, dexterous body and controlling their weight. In addition, infant studies by Koenig et al. (2011) reported that the abundance of *Bacteroidetes* increased after the introduction of peas and other table foods. The higher proportion of *Bacteroidetes* in the gut microbiota of children from Burkina Faso, Africa compared with European children may be due to the typical Western diet containing high levels of protein, sugar and fat while being low in fiber (De Filippo et al. 2010). Moreover, the probability of developing diseases such as cardiovascular disease (Fung et al. 2001; Hu et al. 2000; Heidemann et al. 2008), type 2diabetes (Van Dam et al. 2002; Fung et al. 2004) and mortality by any cause (Heidemann et al. 2008) was comparatively lower in individuals with a “reasonable” diet. Due to the limited availability of small mammals and harsh conditions (Zhang and Ma et al. 1999), the diet of wild sables mainly consists of plants, such as berries and nuts. The reduced abundance of *Bacteroidetes* in housed sables may be due to the increased availability of fish and other animal protein in fur farms. However, previous studies have traditionally shown a decreased abundance of *Bacteroidetes* in vegetarians and vegans compared with those receiving an omnivore diet (Zimmer et al. 2012). Mozaffarian et al. (2011) also reported a negative correlation between weight gain and individuals who change to a mainly plant-based diet. In addition, Costa et al. (2012) found that *Bacteroidetes* are a small proportion of the intestinal bacteria in healthy horses. Thus, the exact role of this phylum and its functional contribution remain unclear, and further studies with an increased sample size should be conducted.

As the primary source of energy, food is essential to growth and development, immunity and self-regulation of life (Dutton and Turnbaugh 2012). Thus, diet has considerable effect on the composition of intestinal bacterial communities. The wild sables (*M. zibellina*) in our study are from the Northeast region of China, where they experience significantly different foraging conditions between summer and winter. In general, the sable diet consists of primarily small rodents, followed by plant food, birds, and occasional insects (Bao et al. 2003). Although sables typically choose foraging sites with an abundance of food (Zhang and Ma 1999), heavy snow in the winter generally creates shortages in meat-based food, such as rodents, containing sufficient protein. As a result, pine nuts and berries, which are rich in fiber and low in fat, usually become their major food source. The production of SCFA (short-chain fatty acids) may increase due to fiber fermentation, and dietary changes would also alter the amount of SCFAs immediately (Rosenberg et al. 2013). However, De Filippo et al. (2010) reported that African groups had significantly less SCFAs compared with European groups. Thus, in addition to measuring the sable microbiome, SCFAs should be considered to be an important physiological and biochemical index for further investigation. In addition, it would be interesting to characterize the intestinal microbiota in wild sables that are temporarily switched to the same diet as housed sables (C1–C14) living in the fur farm. Several studies have indicated that the effects on gut microbiota caused by dietary changes can occur in a short time interval (Turnbaugh et al. 2009a). Because both groups of sables are fed the same diet consisting of primarily fish, eggs, and wheat bran, it would be interesting to examine new fecal samples from the wild sables 3 months post-capture to determine if there is a difference in the levels of *Bacteroidetes*, *Actinobacteria*, and *TM7*. We hypothesize that for most mammals, the diversity of the intestinal bacteria community may change when a new diet is introduced.

In addition, PCoA and NMDS analyses of housed sables with identical dietary and environmental conditions revealed a variability of bacterial diversity that may be due to age, sex and host genetics (Zhang et al. 2010). Although the distinction is subtle, PCoA and NMDS analyses showed that the phylogenetic trees calculated using different methods were consistent with each other. This is another instance where additional fecal samples from wild sables would be beneficial.

Phylogenetic analysis using high-throughput sequencing of bacterial 16S rRNA is very rapid (Woese 1987), but it reveals a limited portion of the gut microbiome compared with traditional culture methods (Rosenberg et al. 2013). Nevertheless, our study suggests that there is a difference in diversity of intestinal bacteria community between wild and housed sables, demonstrating that the methods we used were helpful in evaluating the gut microbiome for this rare animal that resides in China. Our findings suggest that deeper research should be continued to reveal the specific functional and molecular contribution of particular types of microbes to the intestinal microbiome of sables.

Our study characterized differences in the intestinal microbiota of wild and housed sables, and these differences were mainly due to distinctions in diet. *Bacteroidetes* are the predominant phylum in the intestinal microflora of wild sables but represent only a minor proportion in microflora of housed sable, and this suggests that they may play an important role that should be studied further. Because this is the first report of an abundance of *Actinobacteria* in wild sables and the presence *TM7* in housed sables, more research must be conducted. The characterization and comparison of the gut bacterial community indicates that the sable intestinal microbiome is complex, and studying it has implications for wildlife conservation efforts. Moreover, it will be important to perform more studies that characterize how environmental differences directly affect the bacterial populations present in fecal samples.
